# Quality of Life in Teenagers and Adults With Coeliac Disease: From Newly Spanish Coeliac Disease Questionnaire Validation to Assessment in a Population-Based Study

**DOI:** 10.3389/fnut.2022.887573

**Published:** 2022-05-31

**Authors:** María de Lourdes Moreno, Diego Sánchez-Muñoz, Carolina Sousa

**Affiliations:** ^1^Departamento de Microbiología y Parasitología, Facultad de Farmacia, Universidad de Sevilla, Seville, Spain; ^2^Instituto Digestivo, Hospital Sagrado Corazón, Seville, Spain

**Keywords:** coeliac disease, gluten-free diet, quality of life, questionnaire, validation, EQ-5D

## Abstract

**Background:**

Coeliac disease (CD) is an immune-mediated systemic disorder elicited by the ingestion of gluten in genetically predisposed individuals. Gluten restriction in CD sufferers leads to numerous limitations in various aspects of daily life and can significantly impact the quality-of-life (QoL). The specific and widely used Coeliac Disease Questionnaire (CDQ) is an excellent tool to evaluate QoL in patients with CD, assessing physical, psychological, and social domains. This questionnaire is unavailable in Spain. Therefore, our study is the first to translate, culturally adapt, validate, and apply the Spanish version of CDQ to a representative sample of Spanish teenagers and adults with CD.

**Methods:**

A total of 153 CD participants with biopsy-proven and self-reported gluten-free adherence were included in the cross-sectional study, which included four stages: (1) translation and retranslation of the French CDQ version into Spanish; (2) cultural adaptation and semantic evaluation; (3) CDQ validation through the internal consistency determination and reproducibility of the QoL; and (4) application of the questionnaire to Spanish teenagers and adults with CD and estimation of QoL using EQ-5D.

**Results:**

The internal consistency and test–retest reliability of the Spanish CDQ were satisfactory and no ceiling or floor effects were detected. Significant correlations were identified between the CDQ scales, and the instrument for validation covering similar dimensions of the QoL was identified. The mean CDQ total score was 131.03 ± 24.1, and the social domain had the highest rating. There was no correlation between the time spent on a gluten-free diet and QoL. A significantly higher QoL score was reported among males and adolescents in the 15–17 age groups.

**Conclusion:**

The newly Spanish CDQ is an appropriate tool to assess the QoL of the teenager and adult patients with CD. This study highlights the importance of identifying the affected scales to address actions to reduce the impact of the gluten-free diet burden of the coeliac patients and maintain public health regulations that support patients with chronic diseases such as CD.

## Introduction

Coeliac disease (CD) is an immune-mediated systemic disorder elicited by the ingestion of gluten that affects 1% of the world’s population and can develop throughout life (1, 2). CD is triggered by the dietary gluten in genetically predisposed individuals, causing damage to the small intestinal villi, leading to decreased functionality of the intestinal surface and malabsorption of nutrients (3). The cornerstone of treatment for CD is a strict, lifelong, gluten-free diet (GFD). However, it is difficult to maintain a strict oral diet for life because the ubiquitous nature of gluten and the cross-contamination of gluten can occur at many stages of food production, from the factories, and also handcraft enterprises, restaurants, and even in households (4). Therefore, the challenge of GFD leads to numerous limitations in patients with CD in various aspects of daily life, including traveling, shopping, and eating out, which can significantly affect the patient’s quality-of-life (QoL) (5–7). Previous studies have reported a significant psychological impact, anxiety, and depression as ongoing issues in CD (8).

The analysis of patient-reported outcomes in chronic diseases has been proposed as a multidimensional measure of physical and mental health (9). In particular, the analysis of health-related quality-of-life (HRQoL) has attracted growing interest to help clinicians and public health officials understand the holistic burden of diseases, such as CD (10). Moreover, the perception of HRQoL is essential for the evaluation and implementation of measures that can reduce the burden on affected patients. There are two main types, generic and specific questionnaires. Generic questionnaires measure the daily life aspects of HRQoL in patients who have several conditions, whereas specific questionnaires focus on specific aspects related to the disease and its treatment (11). Generic self-administered questionnaires are mostly used as indicators for HRQoL, including the EuroQol 5-Dimensions (EQ–5D) and Short Form-36 Health Survey (SF-36) (12, 13). The EQ–5D is the preferred preference-based instrument to measure HRQoL in several countries as provides a simple and easy-to-use alongside and is readily valued by the general population (14). The EQ–5D describes health status in five dimensions: mobility, self-care, usual activities, pain/discomfort, and anxiety/depression, and converted raw ratings of QoL (15).

The CD-specific QoL questionnaires take into consideration the situational circumstances of adhering to GFD and, for adult patients with CD include the Coeliac Disease Questionnaire (CDQ) (16) and the Coeliac Disease Quality-of-Life (CD–QoL) surveys (17). The CDQ differs from the CD-QoL in that it incorporates a specific domain of gastrointestinal symptoms and focuses on psychological wellbeing (emotions and worries) and social functioning, while the CD–QoL employs a need-based model. However, a questionnaire adaptation to the cultural, socioeconomic, and language environment of the area to be implemented is mandatory to produce semantically equivalent versions and maintain measurement properties across language versions and thereby allow data from different language groups to be compared and/or pooled. To date, in Spain, only CD–QoL has been adapted and validated by comparison with EQ–5D (18). The CDQ original version is from Germany, since then has been adapted and validated in France, Turkey, Italy, Iran, and more recently in Argentina with good psychometric properties (19–23). The CDQ also has been adapted and validated in Brazil and Poland (24, 25). However, all the published studies have used SF-36 as an assessment of HRQoL despite the preferred EQ–5D and did not include an interesting group of adolescents and youth population without close parental supervision, unlike children. Therefore, our study aimed for the first time to translate, culturally adapt, validate, and apply the Spanish CDQ version and estimate the HRQoL using EQ–5D in a representative sample of the Spanish teenagers and adults with CD. We work on the French version of CDQ to Spanish due to its excellent validation value and lifestyle proximity.

## Materials and Methods

### Study Design and Population

**FIGURE 1:**
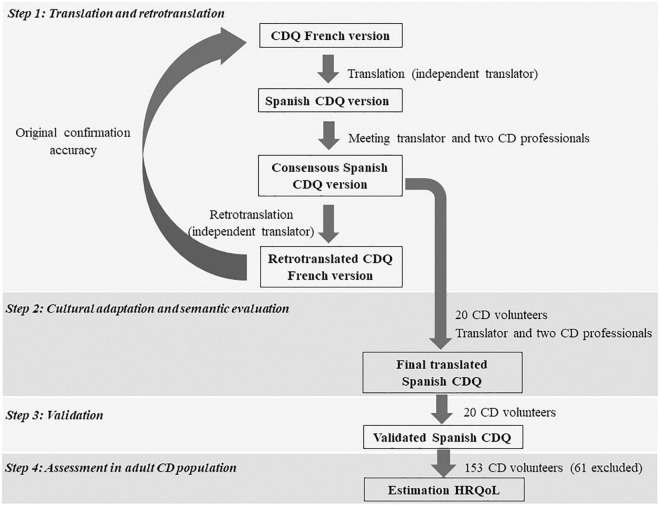
Study design flowchart.

The cross-sectional study involved four defined steps schematized in Figure 1 as follows: (1) translation and retranslation of the French version of CDQ into Spanish; (2) cultural adaptation and semantic evaluation; (3) validation of CDQ through the internal consistency determination and reproducibility of the QoL; and (4) application of the questionnaire to Spanish adult and adolescent patients with CD and statistical analysis. A study that included patients with CD was conducted between October 2020 and September 2021 by the Gastroenterology Unit in two hospitals in southern Spain (Sevilla and Algeciras).

The inclusion criteria were as follows: age > 15 years, biopsy-proven CD diagnosis, and self-reported adherence to a GFD. Demographic characteristics were collected: sex, time on the GFD (< 2, 2–5, and > 2 years), level of education, and professional status (Table 1).

**TABLE 1:** Sociodemographic and medical data of patients.

Variable		*n* (%)
**Age (mean ± SD) in years**	29.83 ± 14.64	
**Age**	15–17 years	20 (21.7)
	18–25 years	24 (26.1)
	26–59 years	42 (45.7)
	<60 years	6 (6.5)
**Sex**	Male	37 (40.2)
	Female	55 (59.8)
**Time on GFD**	<2 years	55 (59.8)
	2–5 years	37 (40.2)
	>5 years	27 (29.3)
**Level of educational attainment**	Primary	5 (5.4)
	Secondary	36 (39.1)
	University	51 (55.4)
**Occupational status**	Employee	35 (38.0)
	Retired	6 (6.8)
	Household	1 (1.1)
	Student	40 (43.5)
	Unemployed	10 (10.9)

*GFD, gluten-free diet.*

The exclusion criteria included the inability to complete all the items questioned and refusing to sign the informed consent form. A total of 153 participants with CD (95 females and 58 males, age range 15–78 years) were randomly enrolled. Ethical approval was obtained from the Regional Ethics Review Board (0504-N-19). All the participants received a debriefing in advance and written consent was obtained from all the adult patients and parents or legal guardians in the case of adolescents.

### Translation and Retranslation

The CDQ was translated from French to Spanish by an external independent translator unrelated to medical staff. After translation, two health professionals specializing in CD met with the translator and integrated views and discrepancies. Subsequently, a retrotranslation of the Spanish questionnaire into French was carried out to confirm its accuracy.

### Cultural Adaptation, Semantic Evaluation, and Validation

The widely used Delphi method was used for cultural adaptation and semantic evaluation. To assess whether the translated questionnaire was interpreted in our geographical, cultural, and social contexts, 20 CD volunteers were recruited in the Digestive Specialties Institute of Algeciras (Spain). The sample size calculation for the pilot study was performed after review of the populations in the previous studies and the minimum proportion in the reference groups were assumed to be 0.04. Accepting an expected difference of 0.06, with an alpha risk of 5%, a beta risk of 20% (80% of statistical power), and a loss rate of 10%, the sample size needed was 20 subjects. The calculation was made using the tool GRANMO v7.12 April 2012 (Institut Municipal d’Investigació Mèdica, Barcelona, Spain). Although these initial questionnaires were not included in the analysis, these patients were also asked to fill out a new questionnaire afterward in the validation phase. The Spanish-translated CDQ version was administered to answer the following questions on each CDQ item: (1) Is the question well understood? (2) Do the questions have a single meaning? and (3) Would you change any words or group of words to provide a better understanding of the question? in which the possible answer was “yes” or “no, providing a free text to explain if the answer was “no.” Finally, a subheading with suggestions is available. Subsequently, the two doctors met again together with the translator to provide the final translated CDQ.

To validate Spanish version of the CDQ, test–retest reliability, internal consistency, and ceiling/floor effects were calculated. The Spearman’s rank correlation coefficient was used to analyze test–retest reliability and the Cronbach’s coefficient alpha was used as an adequate measure of internal consistency. Alpha values between 0.70 and 0.95 were considered acceptable (26). The identification of floor and ceiling effects indicates the ability of the questionnaire to distinguish between respondents at the extreme ends of the scale. Floor and ceiling effects were assessed by dimension and considered as significant (≥ 15%), moderate (10 to < 15%), minor (5 to < 10%), and negligible (< 5%) (27) in relation to the participants scored the highest or lowest possible score.

The Spanish version of the CDQ was held as shown in Figure 1. Although the original version was the German, we chose the French version due to its excellent validation value, as well as its proximity to the Spanish since Mediterranean culture and lifestyle are the opposite of the German’ culture and lifestyle. After various stages, the translator and health professionals provided the final translated CDQ (Supplementary Material).

### Spanish Coeliac Disease Questionnaire Application

The Spanish CDQ version comprising 28 questions was classified into four subscales as follows: gastrointestinal area, emotional, social, and worries. Each of the 28 questions presented a Likert-type answer model with seven possible answers, where score 1 considers “the worst possible state,” while score 7 is “the best possible state.” The maximum overall score was established at 196 points, with a maximum score on each subscale of 49 points (16). The CDQ was sent through Google Forms application.

### The EuroQol 5-Dimensions Survey

The EQ–5D survey comprised five questions on mobility, self-care, ability to perform daily activities, presence of pain or discomfort, and presence of anxiety and depression symptoms with three possible answers for each item (1, no problems; 2, moderate problems; and 3, severe problems). A summary index with a maximum score of 1 can be derived from these five dimensions by conversion to a table of scores. In addition, a visual analog scale (VAS) indicated the general health status, with 100 points indicating the best health status. By scoring on the VAS, patients can be classified as having good QoL (≥ 70), regular (between 50 and 69), and poor (< 50). The EQ–5D survey was sent through Google Forms application.

### Statistical Analysis

All the results are expressed as the mean SD. Cronbach’s alpha and Spearman’s correlation coefficients were used to assess the internal consistency and test–retest reliability, respectively. Pearson’s chi-square test was used to assess the agreement between the CDQ score and the EQ–5D survey. For intraindividual comparisons, differences of ≥ 12 within the total score and ≥ 3 within each subscore can be regarded as a minimum important clinical difference. For comparison of groups in all statistical tests, a *p*-value of less than 0.05 was considered statistically significant. Statistical analyses were performed using IBM SPSS 25.0 version (IBM Corporation, Armonk, NY, United States).

## Results

### Acceptance and General Characteristics of the Subjects

Of the 153 participants with CD who were initially enrolled in the study and signed informed consent, 61 were excluded due to not meeting the eligibility criteria or partially completing both questionnaires CDQ and EQ–5D (46 lack one question, nine participants lack two questions, and six incomplete questionnaires concurred with patients with three or more unfilled questions). None of the questionnaires was rejected due to multiple responses. The profiles of the excluded respondents more frequently were females, aged 26–59 years, and on GFD for < 2 years.

We included a total of 92 participants (55 [59.8%] females and 37 [40.2%] males), with an average age of 29.8 ± 14.6 years (range, 15–78 years) in the analysis. As shown in Table 1, homogeneous groups for the study CD cohorts were attempted in relation to the distribution of the volunteers by sex, time of CD diagnosis, and time on GFD.

### Spanish Coeliac Disease Questionnaire Validation

The Spanish version of the CDQ followed the flowchart held in Figure 1. The descriptive statistical analysis was presented using subscales and the sum and mean of the scores were examined. The 20 patients did not mention any possible change to the initial Spanish translation. Test–retest reliability for each subscale of the questionnaire for the first 20 patients with CD who responded to the questionnaire was calculated and obtained a score of 0.99 on each of the subscales (Table 2). According to the international agreement, the reliability value is indicated as excellent (1–0.8), good (> 0.6), moderate (> 0.4), and poor (< 0.4) (28), therefore, our results indicate excellent reliability. The internal consistency of all the subscales was verified by the Cronbach’s alpha, yielding a score of 0.83–0.92 (Table 2), indicating a good internal consistency as internal consistency is indicated as 1 < α ≥ 0.9, excellent; 0.7 ≤ α < 0.9, good; 0.6 ≤ α < 0.7, acceptable; 0.5 ≤ α < 0.6, poor; and α < 0.5, unacceptable (29). A comparison of the internal consistency of this study was attempted with all translated and validated CDQs according to the domains. The Cronbach’s alpha was the highest in the Spanish CDQ in the worries domain and remained among the best in the social and gastrointestinal domains, except for the emotion. Similarly, the ceiling and floor effects of the subscales were established, ranging from 0.51 to 3.11% and 0.13 to 1.06%, respectively, and were therefore considered negligible (Table 2).

**TABLE 2:** Reliability and precision of the subscales of the Spanish CDQ validation.

Subscale	Sum	Mean (standard deviation)	Ceiling effect (%)	Floor effect (%)	Internal consistency (Cronbach’s alpha)	Test-retest reliability (Spearman’s correlation)
**Gastrointestinal**	32.53 ± 7.76	4.64 ± 1.11	1.41	0.53	0.830	0.99
**Emotional**	27.48 ± 4.78	3.92 ± 0.68	0.51	0.23	0.852	0.99
**Social**	40.23 ± 5.84	5.74 ± 0.83	3.11	0.13	0.850	0.99
**Worries**	30.79 ± 5.72	4.39 ± 0.71	1.96	1.06	0.923	0.99

*CDQ, Coeliac Disease Questionnaire.*

**FIGURE 2:**
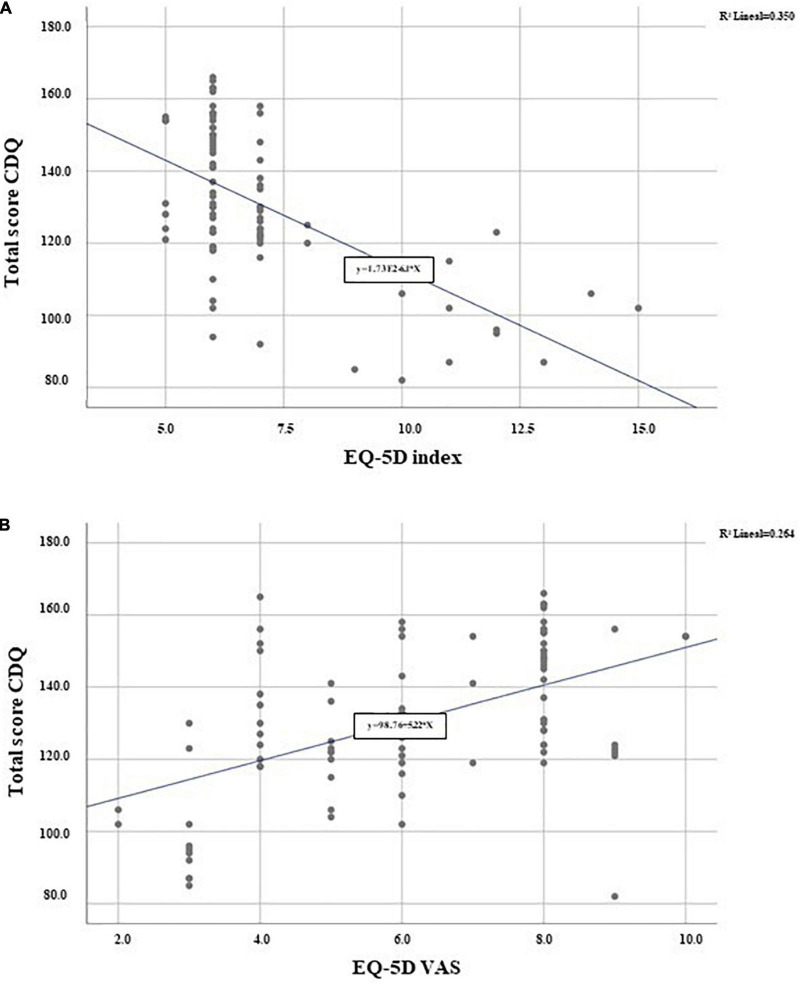
Scatter plot of the CDQ mean score and EQ-5D survey mean score. **(A)** EQ-5D index score *r* = −0.592; *p* < 0.01; **(B)** EQ-5D VAS *r* = 0.514; *p* < 0.01.

The comparison between the EQ–5D visual scale and the Spanish CDQ was also performed. Mobility, personal self-care, ability to accomplish daily activities, pain, and anxiety/depression symptoms scores were worse in the coeliac patients with the worst scores on the CDQ, and these results were consistent across all the four subscales (Table 3). Furthermore, higher consistency was added by the Pearson’s coefficient correlation between the total score of the study questionnaire and the total score of the validated Spanish version of the EQ–5D QoL questionnaire (*r* = −0.592; *p* < 0.01) and the score obtained on the visual scale of this questionnaire (*r* = 0.514; *p* < 0.01) (Figures 2A,B).

**TABLE 3:** Subscores of the Spanish CDQ are subcategorized by the characteristics of the participants.

Variable	Character	*n*	Gastrointestinal			Emotional			Social			Worries		
			Sum	Media	*p*	Sum	Media	*P*	Sum	Media	*p*	Sum	Media	*P*
**Sex**	Men	37	34.21 ± 0.77	4.88 ± 0.96	**0.088**	29.27 ± 4.18	4.18 ± 0.59	**0.003**	41.78 ± 5.62	5.96 ± 0.80	**0.036**	31.51 ± 4.74	4.50 ± 0.67	**0.262**
	Women	55	31.40 ± 8.22	4.48 ± 1.17		26.27 ± 4.82	3.75 ± 0.68		39.18 ± 5.80	5.59 ± 0.83		30.31 ± 5.19	4.32 ± 0.74	
**Age**	Teenager	20	40.40 ± 3.33	5.77 ± 0.47	**<0.01**	31.95 ± 2.41	4.56 ± 0.34	**<0.01**	46.55 ± 2.18	6.65 ± 0.31	**<0.01**	35.05 ± 2.45	5.01 ± 0.35	**<0.01**
	Joung	24	36.83 ± 5.33	5.26 ± 0.76		30.04 ± 2.98	4.29 ± 0.42		42.75 ± 3.47	6.10 ± 0.49		33.45 ± 4.45	4.78 ± 0.63	
	Adult	42	28.35 ± 4.69	4.05 ± 0.67		25.07 ± 3.57	3.58 ± 0.51		37.52 ± 3.71	5.36 ± 0.53		27.47 ± 3.58	3.92 ± 0.51	
	Older	6	18.33 ± 4.13	2.61 ± 0.59		19.16 ± 2.48	2.73 ± 0.35		28.01 ± 1.78	4.00 ± 0.25		29.16 ± 6.14	4.16 ± 0.87	
**+Time on GFD**	<2 years	55	31.40 ± 8.22	4.48 ± 1.17	**0.229**	26.27 ± 4.82	3.75 ± 0.68	**0.012**	39.18 ± 5.81	5.59 ± 0.82	**0.082**	30.31 ± 5.19	4.33 ± 0.74	**0.533**
	2–5 years	10	34.70 ± 4.87	4.95 ± 0.69		29.40 ± 3.41	4.20 ± 0.48		43.00 ± 4.64	6.14 ± 0.66		31.40 ± 3.43	4.48 ± 0.49	
	>5 years	27	34.03 ± 7.43	4.86 ± 1.06		29.22 ± 4.49	4.17 ± 0.64		41.33 ± 5.96	5.90 ± 0.85		31.55 ± 5.19	4.50 ± 0.74	
**Mobility**	No problems	80	34.38 ± 6.32	4.91 ± 0.90	**<0.01**	28.66 ± 3.81	4.09 ± 0.54	**<0.01**	41.62 ± 4.64	5.94 ± 0.66	**<0.01**	31.18 ± 4.94	4.45 ± 0.71	**<0.01**
	Some problems	12	28.66 ± 3.61	2.88 ± 0.62		19.58 ± 2.64	2.79 ± 0.37		30.91 ± 4.91	4.41 ± 0.61		28.16 ± 4.96	4.02 ± 0.71	
**Personal care**	No problems	82	33.96 ± 6.81	4.85 ± 0.97	**<0.01**	28.36 ± 4.22	4.05 ± 0.60	**<0.01**	41.30 ± 5.02	5.90 ± 0.71	**<0.01**	31.01 ± 5.01	4.43 ± 0.71	**0.142**
	Some problems	8	20.12 ± 4.48	2.87 ± 0.54		20.00 ± 2.39	2.85 ± 0.34		35.75 ± 3.81	4.39 ± 0.54		27.75 ± 4.83	3.96 ± 0.69	
	Unable	2	23.50 ± 4.95	3.35 ± 0.70		21.00 ± 2.82	3.00 ± 0.40		34.00 ± 8.48	4.85 ± 1.21		34.00 ± 1.41	4.85 ± 0.20	
**Daily activity**	No problems	66	34.86 ± 6.27	4.98 ± 0.89	**<0.01**	28.97 ± 3.64	4.13 ± 0.52	**<0.01**	42.15 ± 4.42	6.02 ± 0.63	**<0.01**	31.54 ± 4.93	4.50 ± 0.70	**0.071**
	Some problems	23	27.47 ± 8.22	3.92 ± 1.17		24.17 ± 5.45	3.45 ± 0.78		36.17 ± 6.14	5.16 ± 0.87		28.82 ± 4.51	4.11 ± 0.64	
	Unable	3	20.00 ± 3.00	2.85 ± 0.42		20.00 ± 1.00	2.85 ± 0.14		29.00 ± 1.00	4.14 ± 0.14		29.33 ± 8.14	4.19 ± 1.16	
**Pain discomfort**	None	55	36.87 ± 5.37	5.26 ± 0.76	**<0.01**	29.98 ± 3.13	4.28 ± 0.44	**<0.01**	43.09 ± 4.33	6.15 ± 0.61	**<0.01**	32.81 ± 4.35	4.68 ± 0.62	**<0.01**
	Moderate	35	26.34 ± 6.17	3.76 ± 0.88		23.97 ± 4.42	3.42 ± 0.63		36.40 ± 5.01	5.20 ± 0.71		27.42 ± 4.30	3.91 ± 0.61	
	High	2	21.50 ± 2.12	3.07 ± 0.30		20.00 ± 1.41	2.85 ± 0.20		28.50 ± 0.71	4.07 ± 0.10		34.00 ± 1.41	4.85 ± 0.20	
**Anxiety/depression**	None	64	35.34 ± 5.86	5.04 ± 0.83	**<0.01**	29.03 ± 3.50	4.14 ± 0.50	**<0.01**	42.09 ± 4.18	6.01 ± 0.59	**<0.01**	32.01 ± 4.69	4.57 ± 0.67	**<0.01**
	Moderate	22	27.81 ± 7.93	3.97 ± 1.13		25.36 ± 5.26	3.62 ± 0.75		37.59 ± 6.83	5.37 ± 0.97		28.27 ± 4.39	4.03 ± 0.62	
	High	6	19.83 ± 2.31	2.83 ± 0.33		18.66 ± 1.63	2.66 ± 0.23		30.00 ± 2.09	4.28 ± 0.29		27.00 ± 6.03	3.85 ± 0.86	
**Occupational status**	Employee	35	28.71 ± 4.84	4.10 ± 0.69	**<0.01**	25.37 ± 3.71	3.59 ± 0.53	**<0.01**	35.40 ± 7.19	5.05 ± 1.02	**<0.01**	26.20 ± 3.49	3.74 ± 0.49	**<0.01**
	Retired	6	18.33 ± 4.13	2.69 ± 0.59		19.16 ± 2.48	2.73 ± 0.35		41.80 ± 6.18	5.97 ± 0.88		31.50 ± 5.30	4.50 ± 0.75	
	Household	1	30	4.28		28	4		39.58 ± 5.16	5.65 ± 0.73		30.74 ± 4.77	4.39 ± 0.68	
	Student	40	39.00 ± 4.67	5.57 ± 0.66		31.05 ± 2.86	4.43 ± 0.41		42.58 ± 4.87	6.08 ± 0.69		32.82 ± 4.61	4.69 ± 0.66	
	Unemployed	10	28.80 ± 4.80	4.11 ± 0.68		26.20 ± 3.96	3.74 ± 0.56		39.74 ± 4.78	5.67 ± 0.68		29.25 ± 4.64	4.18 ± 0.66	
**Level of education**	Middle school graduate	5	25.60 ± 10.99	3.65 ± 1.57	**0.05**	23.40 ± 5.59	3.34 ± 0.79	**0.074**	36.75 ± 6.71	5.25 ± 0.96	**0.034**	29.04 ± 5.01	4.14 ± 0.71	**0.085**
	High school graduate	36	34.22 ± 7.83	4.88 ± 1.11		28.41 ± 5.39	4.06 ± 0.77		38.24 ± 5.15	5.46 ± 0.73		29.08 ± 4.15	4.15 ± 0.59	
	College graduate	51	32.01 ± 7.06	4.57 ± 1.01		27.21 ± 4.06	3.88 ± 0.58		40.97 ± 5.94	5.85 ± 0.85		31.43 ± 5.19	4.49 ± 0.74	
**Visual scale**	Good	41	36.17 ± 6.61	5.16 ± 0.94	**<0.01**	29.82 ± 3.59	4.26 ± 0.51	**<0.01**	35.40 ± 7.19	5.05 ± 1.02	**<0.01**	26.20 ± 3.49	3.74 ± 0.49	**0.002**
	Fair	27	31.25 ± 5.48	4.46 ± 0.78		26.59 ± 3.51	3.79 ± 0.50		41.80 ± 6.18	5.97 ± 0.88		31.50 ± 5.30	4.50 ± 0.75	
	Bad	24	27.75 ± 8.89	3.96 ± 1.27		24.45 ± 5.83	3.49 ± 0.83		39.58 ± 5.16	5.65 ± 0.73		30.74 ± 4.77	4.39 ± 0.68	
**Tobacco**	Smoker	25	30.20 ± 7.53	4.31 ± 1.07	**0.078**	25.56 ± 4.91	3.65 ± 0.70	**0.018**	42.58 ± 4.87	6.08 ± 0.69	**0.046**	32.82 ± 4.61	4.69 ± 0.66	**0.045**
	Non-smoker	67	33.40 ± 7.71	4.77 ± 1.10		28.19 ± 4.57	4.02 ± 0.65		39.74 ± 4.78	5.67 ± 0.68		29.25 ± 4.64	4.18 ± 0.66	

*CDQ, Coeliac Disease Questionnaire; GFD, gluten-free diet. The bold type is the result of the statistical probability to make it quicker to visualize for the reader.*

### Application and Analysis of the New Coeliac Disease Questionnaire in the Spanish Adult and Adolescent Coeliac Population

A total of 153 participants with CD received the specific CDQ and general EQ–5D through Google Forms application and signed an informed consent form. The common factor of all the patients with CD included in the study was the maintenance of a GFD. However, previous studies have observed differences in the adherence to GFD in different age groups; therefore, in this study, the respondents were divided into age groups: adolescent (15–17 years), young (18–25 years), adults (26–59 years), and older (> 60 years). Similarly, the time from diagnosis (< 2 and > 2 years) and the level of education (primary, secondary, and university) have been found to be associated with compliance and, therefore, with the influence of subsequent complications. All these data were compared based on the CDQ score in each subscale. Similarly, data on mobility, daily activities, personal care, presence of pain or discomfort, presence of symptoms of anxiety and/or depression, smoking, level of education, and current work activity were compared. Finally, the score data on the EQ–5D visual QoL scale were examined by comparison with the scores on the different subscales of the Spanish CDQ (Table 3).

Males showed higher scores than females in all domains; however, they were only statistically significant in the emotional and social domains (*p* = 0.003 and *p* = 0.036, respectively). The results related to age are noteworthy, as on the four subscales, a lower score was obtained in the older group than in the teens (*p* < 0.01). Despite newly diagnosed coeliacs displaying a short period of GFD, a lower score was obtained on all the scales, and no statistically significant results were obtained except for emotional (*p* = 0.012), with low scores obtained in patients who had been on GFD for < 2 years compared with those of 2–5 and > 5 years on GFD.

The effect of employment status was analyzed in four domains, with the highest scores in students and the worst scores in retired people (*p* < 0.01); however, similar scores were obtained for the different items among employed and unemployed participants. We analyzed participants according to their level of education, as the level of education could influence education on the disease, available gluten-free products, or proper food labeling, and therefore, could improve compliance and QoL. Statistically significant results were obtained only in the social domain (*p* = 0.034), with the highest score for those with secondary education and the lowest for those with primary education.

Patients with CD who are active smokers showed the worst results on all the subscales compared with those without a history of smoking. However, the gap between these subgroups of patients’ analysis was less demonstrative in the gastrointestinal symptom subscale than in the other domains (33.40 ± 7.71 vs. 30.20 ± 7.53; *p* = 0.078).

In addition, the interesting results obtained in all domains in the teenager group led to a more in-depth study to examine their HRQoL, and even the onset, sex, and time on GFD affected the health self-valuation. Females had worse scores on all the subscales, reaching statistically significant differences in the emotional and social subscales. Patients with scores: < 50 on the EQ–5D visual scale had a significantly lower score on the gastrointestinal symptom’s subscale than those with scores of > 50 (41.33 ± 2.51 vs. 34.00 ± 7.07; *p* < 0.01). However, there were no significant differences in scores on the different subscales depending on the time they had been on the GFD (Table 4).

**TABLE 4:** Subscores of the coeliac teenagers in the Spanish CDQ.

			Gastrointestinal			Emotional			Social			Worries		
		*n*	Suma	Media	*P*	Suma	Media	*p*	Suma	Media	*p*	Suma	Media	*P*
**Sex**	Men	11	41.00 ± 1.89	5.85 ± 0.27	0.388	33.00 ± 2.04	4.71 ± 0.29	0.027	47.54 ± 1.50	6.79 ± 0.21	0.02	35.63 ± 1.68	5.09 ± 0.24	0.249
	Women	9	39.66 ± 4.55	5.66 ± 0.65		30.66 ± 2.29	4.38 ± 0.32		45.33 ± 2.34	6.47 ± 0.33		34.33 ± 3.12	4.90 ± 0.44	
**Time on GFD**	<2 years	9	39.66 ± 4.65	5.66 ± 0.65	0.532	30.66 ± 2.29	4.38 ± 0.32	0.08	45.33 ± 2.34	6.47 ± 0.33	0.061	34.33 ± 3.12	4.90 ± 0.44	0.35
	2–5 years	4	40.00 ± 0.81	5.71 ± 0.11		32.50 ± 1.73	4.64 ± 0.24		48.00 ± 0.01	6.85 ± 0.01		34.75 ± 0.50	4.96 ± 0.07	
	>5 years	7	41.57 ± 2.14	5.93 ± 0.30		33.28 ± 2.28	4.75 ± 0.32		47.28 ± 1.88	6.75 ± 0.28		36.14 ± 1.95	5.16 ± 0.27	
**Visual scale**	Good	15	41.06 ± 2.01	5.88 ± 0.28	<0.01	32.20 ± 2.14	4.60 ± 0.31	0.655	46.40 ± 2.22	6.62 ± 0.31	0.419	35.33 ± 2.71	5.04 ± 0.38	0.476
	Fair	3	41.33 ± 2.51	5.90 ± 0.35		31.66 ± 0.57	4.52 ± 0.08		48.00 ± 0.01	6.85 ± 0.01		35.00 ± 0.01	5.00 ± 0.01	
	Bad	2	34.00 ± 7.07	4.85 ± 1.01		30.50 ± 6.36	4.35 ± 0.91		45.50 ± 3.53	6.50 ± 0.49		33.00 ± 1.41	4.71 ± 0.20	

*CDQ, Coeliac Disease Questionnaire; GFD, gluten-free diet.*

Of the validated CDQs, the acceptance of the Spanish CDQ was like that of the French and Italian CDQs, although the number of patients included in this study was lower, with the Turkish and Iranian questionnaires having the lowest number of patients included and the German and French questionnaires having the highest number. However, this study was the most homogeneous in terms of sex and remarkable for its teenager inclusion.

## Discussion

The ubiquitous nature of gluten constitutes a global problem for coeliacs because the GFD restricts the choice of many foods, with a significant impact on the behavioral, emotional, and psychosocial domains of coeliacs (7, 30). Assessing the HRQoL has been increasingly acknowledged in research and clinical studies in CD, thus, contributing to improved interventions and measures that can reduce the burden on the affected patients. The benefit of using CD-specific questionnaires compared with the generic QoL is that the questions included are specific to the disease, the GFD, and their limitations. Although specific QoL questionnaires for adult coeliacs have been used successfully (16, 17), the adaptation of the questionnaire to the cultural, socioeconomic, and language environment of the area in which it will be implemented is a key issue. The CDQ questionnaire, which mainly focuses on symptoms and decreased daily function, has been translated and validated in different languages. However, no cultural Spanish version has been available to date. In this study, we developed for the first time a new valid and specific CDQ for patients with CD in Spanish and estimated the HRQoL, including a representative sample of teenagers and adults with CD by comparison with EQ–5D.

The CDQ was translated with a rigorous methodology, providing safeguards against subjectivity, and ensuring equivalence between the original French language questionnaire and the Spanish translation. The internal consistency and test–retest reliability of the Spanish CDQ were satisfactory, and no ceiling or floor effects were detected from the inputs in 20 participants, as recommended by Devellis (31). The comparison of internal consistency between all the available translated CDQs showed that Cronbach’s alpha was the highest in the Spanish CDQ in the worries domain and remained among the top in other domains except for the emotional. All the four domains of the CDQ indicated good internal consistency (Cronbach’s alpha > 0.7). Furthermore, a significant and strong correlation was identified between the Spanish CDQ and EQ–5D visual scale; therefore, it is a valid and reliable instrument with good acceptability and feasibility to assess the QoL of CD populations in clinical and research settings.

Although there was a female predominance in CD, we made every effort to achieve male recruitment in the study groups to reach the most homogeneous percentage of females (59.8%) and males (40.2%) of all CDQ validation studies published, in which men did not exceed 30% representation. All the studies on HRQoL of adults with CDQ have included adults (15, 18–22), but no studies included teenagers. In this study, we have examined how adolescents with CD valued their present HRQoL.

Our results indicate that males had higher scores for the CDQ on all the subscales than females; according to previous studies, which showed that females with CD experience a lower level of QoL than males, reporting more distress caused by daily life restrictions and perceiving a higher burden of CD than males (32–35). Regarding age, evidence from the general population across countries shows that QoL decreases with age (36), which may be due to other related concerns and physical conditions of older people, and that the adult coeliacs adhering to GFD have a negative impact on HRQoL, especially in the social domain (5, 37). Our findings indicate that the extent of gluten restriction affects multiple aspects of daily life in the older group. According to Lee et al. (38), length of time on a GFD was associated with higher social and emotional measures compared to those on a GFD for < 2 years.

A more detailed study of the teenager population determined that sex-influenced QoL since adolescent females showed a lower score than males on the emotional and social subscales. No relationship was found between time on the GFD and QoL. Unlike the rest of the population, it is striking how the scores are very high on the social subscale regardless of the time they have been doing GFD, which can indicate that coeliac teenagers are not greatly affected from the social point of view to perform strict GFD. Currently, the absence of symptoms after consuming a small amount of gluten is the most common cause of diet failure in teenagers (39). Although adherence to GFD was not evaluated in our study because it was not within the objectives, it could be interesting to extend studies with a greater number of coeliacs to evaluate the adherence to GFD and QoL relationship in this interesting group. Data obtained in teens showed that QoL measurements should be differentiated according to age, and therefore, teens should not be included in the same group as other adults when asked about the implications of CD in their lives. This may lead to new studies that focus specifically on assessing teenagers’ QoL with specific instruments that also provide valuable insight into the potential adverse of impacts parental QoL.

Finally, to strengthen the reliability of the study, we compared the specific Spanish CD questionnaire with the global EQ–5D widely used in several diseases and even in CD (32). Particularly, EQ–5D has been used to assess the validation and transcultural adaptation of other CD-specific questionnaires, such as the CD–QoL survey (16) although most have compared the generic QoL questionnaire SF-36 (18–20, 25). We decided to use the EQ–5D due to its simplicity and easy understanding, and because the VAS provides a very close to reality approach to the general status of the patient. The total score and the VAS were significant according to the Pearson’s chi-square test, showing that the responses of the Spanish patients in the CDQ were accordingly associated with their QoL.

Therefore, the results of this study can help to design and implement effective and sustainable interventions to support coeliacs with excessive burden and stress to prevent poor QoL outcomes and arrange psychological support if required.

## Conclusion

This study is the first time to provide a validated Spanish CDQ for assessing QoL in adults and teenagers with CD. It is a valuable tool in the follow-up of patients in the hospitals to identify and address the most deteriorated areas, opening a wide range of actions to reduce the impact of the GFD on patients with CD.

## Data Availability Statement

The original contributions presented in the study are included in the article/Supplementary Material, further inquiries can be directed to the corresponding author/s.

## Ethics Statement

The studies involving human participants were reviewed and approved by the Ethics Committee of Sevilla Sur, Spain (0504-N-19). Written informed consent to participate in this study was provided by the participants’ legal guardian/next of kin.

## Author Contributions

MLM and DS-M conceptualized the study, wrote, and prepared the original draft. MLM, DS-M, and CS contributed to the methodology and formal analysis, reviewed, and edited the manuscript. All authors have read and agreed to the published version of the manuscript.

## Conflict of Interest

The authors declare that the research was conducted in the absence of any commercial or financial relationships that could be construed as a potential conflict of interest.

## Publisher’s Note

All claims expressed in this article are solely those of the authors and do not necessarily represent those of their affiliated organizations, or those of the publisher, the editors and the reviewers. Any product that may be evaluated in this article, or claim that may be made by its manufacturer, is not guaranteed or endorsed by the publisher.
