# Supplementary material for: Quality of Life in Teenagers and Adults With Coeliac Disease: From Newly Spanish Coeliac Disease Questionnaire Validation to Assessment in a Population-Based Study
Source: Front Nutr. 2022 May 31;9:887573. doi: 10.3389/fnut.2022.887573 (PMC9194896; doi:10.3389/fnut.2022.887573)
Supplement: Supplementary file 1 [file Data_Sheet_1.docx]

**Spanish version of the Celiac Disease Questionnaire (CDQ)**

Nos gustaría saber cómo se ha sentido en las dos últimas semanas. Este cuestionario trata sobre los síntomas de la celiaquía, sobre cómo se siente en general y sobre su estado de ánimo.

Lea atentamente cada pregunta y escoja la respuesta (solo una) que mejor describa su situación en las dos últimas semanas.

1. En estas dos últimas semanas, ¿se ha sentido molesto por una necesidad urgente de evacuar?
2. En estas dos últimas semanas, ¿se ha sentido físicamente cansado/a?
3. En estas dos últimas semanas, ¿se ha sentido nervioso/a o irritable?
4. En estas dos últimas semanas, ¿ha evitado o rechazado una invitación para comer en casa de unos amigos o de la familia por causa de la celiaquía?
5. En estas dos últimas semanas, ¿sus heces han sido blandas o incluso líquidas?
6. En estas dos últimas semanas, en general, ha tenido:
7. En estas dos últimas semanas, ¿ha estado preocupado/a por la idea de haber transmitido o de poder transmitir la celiaquía a sus hijos?
8. En estas dos últimas semanas, ¿ha tenido dolor o calambres en el vientre?
9. En estas dos últimas semanas, ¿ha tenido problemas o dificultad para realizar actividades de ocio o deportes debido a la celiaquía?
10. En estas dos últimas semanas, en general, ¿se ha sentido desmotivado o deprimido?
11. En estas dos últimas semanas, ¿ha tenido hinchazón de vientre o gases?
12. Las personas celíacas tienen, a veces, inquietudes relacionadas con la enfermedad. En estas dos últimas semanas, ¿se ha sentido nervioso/a o angustiado/a con la idea de desarrollar un cáncer a causa de la celiaquía?
13. En estas dos últimas semanas, ¿ha tenido la impresión de que no ha evacuado lo suficiente?
14. En estas dos últimas semanas, ¿se ha sentido tranquilo/a y relajado/a?
15. En estas dos últimas semanas, ¿se ha sentido diferente a los demás o excluido/a a causa de la celiaquía?
16. En estas dos últimas semanas, en general, ¿se ha sentido desestabilizado o a punto de llorar?
17. En estas dos últimas semanas, ¿se ha sentido molesto/a por ganas de eructar?
18. En estas dos últimas semanas, ¿ha sentido molestias en su actividad sexual a causa de la celiaquía?
19. En estas dos últimas semanas, ¿ha tenido náuseas o ganas de vomitar?
20. En estas dos últimas semanas, ¿se ha sentido incomprendido por su familia o amigos cercanos en relación con la celiaquía?
21. En estas dos últimas semanas, se ha sentido:
22. En estas dos últimas semanas, ¿se ha sentido incomprendido por sus compañeros de trabajo o por sus superiores en relación con la celiaquía?
23. En estas dos últimas semanas, ¿se ha sentido penalizado en los estudios o en la carrera profesional por la enfermedad celíaca?
24. En estas dos últimas semanas, ¿se ha sentido molesto por el gasto extra o el tiempo empleado de la dieta sin gluten?
25. En estas dos últimas semanas, ¿ha tenido inconvenientes por problemas reembolso de gastos por alimentos sin gluten u otros tratamientos para la enfermedad celíaca (seguridad social o seguro)?
26. En estas dos últimas semanas, ¿ha sentido una falta de conocimiento de la enfermedad celíaca por parte de los médicos que le han atendido?
27. En estas dos últimas semanas, ¿ha estado preocupado/a por la idea de que la celiaquía le haya sido diagnosticada demasiado tarde?
28. En estas dos últimas semanas, ¿ha estado nervioso/a por los exámenes médicos necesarios por la enfermedad celíaca (análisis de sangre o endoscopia digestiva)?

*Modalidad de respuesta:*

**Preguntas 1-5,7,8,10-17,19,20,22-28**: (1) Todo el tiempo, (2) la mayor parte del tiempo, (3) a menudo, (4) de vez en cuando, (5) pocas veces, (6) casi nunca, (7) nunca

**Pregunta 6:** (1) nada de energía, (2) muy poca energía, (3) poca energía, (4) la energía de siempre, (5) bastante energía, (6) con mucha energía, (7) lleno/a de energía.

**Pregunta 9:** (1) imposibilidad de realizar actividades, (2) gran dificultad, (3) bastante dificultad, (4) alguna dificultad, (5) poca dificultad, (6) casi nada de dificultad, (7) ninguna dificultad, la enfermedad celíaca no ha restringido mis actividades de ocio o deportivas.

**Pregunta 18:** (1) no he tenido actividad sexual a causa de la celiaquía, (2) muy fuertes molestias (3) muchas molestias (4) molestias (5) pocas molestias (6) casi nada de molestia (7) ninguna molestia/ no aplica

**Pregunta 21:** (1) insatisfecho/a o infeliz la mayor parte del tiempo (2) generalmente insatisfecho/a o infeliz (3) bastante a menudo insatisfecho/a o infeliz (4) ni satisfecho/a ni insatisfecho/a (5) bastante a menudo satisfecho/a y feliz (6) generalmente satisfecho/a y feliz (7) la mayor parte del tiempo muy satisfecho/a y feliz.
